# The Prevalence of Human T-Cell lymphotropic Virus Type 1 in Pregnant Women and Their Newborns

**DOI:** 10.5402/2012/975135

**Published:** 2012-11-14

**Authors:** A. Hamedi, F. Akhlaghi, Z. Meshkat, M. Sezavar, H. Nomani, M. Meshkat

**Affiliations:** ^1^Faculty of Medicine, Mashhad University of Medical Sciences, Mashhad 9137119139, Iran; ^2^Women Health Research Center, Faculty of Medicine, Mashhad University of Medical Sciences, Mashhad 9185779613, Iran; ^3^Microbiology and Virology Research Center, Faculty of Medicine, Mashhad University of Medical Sciences, Mashhad 9176699199, Iran; ^4^Department of Biostatistics, Islamic Azad University, Mashhad Branch, Mashhad 9176699199, Iran

## Abstract

The prevalence of HTLV1 virus antibodies was determined in pregnant women and their neonates in Mashhad, northeast of Iran, as shown in this prospective cross-sectional study. 407 women who were hospitalized for delivery participated in this study. Venous blood sampling of pregnant women and umbilical cord of their neonates was done. The first samples of all women were tested for HTLV1 seropositivity by ELISA test and confirmed by PCR method. Then, the presence of HTLV1 in samples of umbilical cords blood in neonates who were delivered to an HTLV1-positive mother was determined by PCR method. All HTLV1-positive infants were called again at the age of 9–12 months, and PCR test was done using HTLV1-specific primers for them. Of all the participating women, 6 persons were HTLV1 seropositive by ELIZA test which was confirmed by PCR test. HTLV1 antibodies were found in cord blood samples by PCR test in 6 newborns who were born to HTLV1-seropositive women. All the six infants at the age of 9–12 months showed positive PCR results by HTLV1 LTR-specific primers; however, only one of them was PCR positive using HTLV1 TAX-specific primers. The prevalence of HTLV1 antibodies in pregnant women was 1.5%, and the vertical transmission rate to their neonates was 16.6%.

## 1. Introduction

Human T-cell lymphotropic virus type 1 (HTLV1) is a retrovirus which can be about 5% of those infected and will develop clinical diseases [1]. The virus infects about 10 to 20 million people worldwide, and it is endemic in some regions such as southern Japan, parts of the Caribbean, South America, the Middle East, and some parts of sub-Saharan Africa [2]. HTLV1 transmission is related to the birth in endemic areas or sexual contact with individuals linked to endemic areas [3]. In endemic areas, the prevalence is varied from 3% to 5% in Trinidad to 30% in Southern Miyazaki, Japanese [4, 5]. In contrast, in nonendemic areas such as the USA and Europe, the prevalence is less than 1% [3]. First, the disease was reported in 1986 in Iran. The most infected subjects were reported from Khorasan province, and the prevalence was different (1% to 3%) in the studies. 

Intrauterine HTLV1 transmission during childbirth causes less than 5% of vertical transmission, and if breastfeeding was done, transmission increases up to 25% [3]. Vertical transmission of HTLV1 infection occurs mainly via mother's milk, and in breastfeeding longer than 6 months, transmission risk is to be 3-fold or more [6]. 

There is no gold standard test to detect HTLV1. Existing diagnostic methods are based on serological tests that contained antibodies against the virus. The most common screening test is the ELISA test which measured antibodies against the viral proteins HTLV1 and HTLV2. This test has high sensitivity but poor specificity due to cross-reacting with HTLV2 because there is a great similarity between the structural proteins of two viruses. The number of false-positive reactions may be due to cross-reacting with anti-HLA antibodies, and this problem is solved by using techniques such as Western blot analysis [7]. Western blot analysis as a confirmatory test is used against both virus gene products (env and gag). The result of ELISA test which is confirmed by Western blot test is used for detection of HTLV1 antibodies [8]. So Western blot analysis can be differentiated between infection with HTLV1 and HTLV2 [9]. Polymerase chain reaction (PCR) is based on proviral DNA extraction of peripheral blood mononuclear cells (PBMCs). This test can also differentiate HTLV1 from HTLV2 that this test can also determine proviral load in the blood. Since PCR test can determine directly DNA provirus, the method is considered as a reference method for determination of infection status, validity of serological methods, and distinguishing between infection with HTLV1 and HTLV2. As the mothers' antibodies are able to pass to neonates and laboratory diagnosis on the neonate sera is not reliable, the PCR method is a useful tool for detecting the HTLV infection in infants who were delivered from HTLV-positive mothers. In addition, PCR for detection of virus infection in the time between exposure and changes in serum can be useful [10]. The aim of this study was to determine the prevalence of HTLV1 virus antibodies in pregnant women and the virus infection in their neonates in Mashhad, Iran.

## 2. Material and Methods

This prospective, cross-sectional study was performed from 15 February 2010 to 15 March 2011 in Omolbanin Hospital, Mashhad, Iran. In this study which was approved by the ethical committee of Mashhad University of Medical Sciences 407 pregnant women participated. Sampling was purposive and convenient as enrolled by women who were hospitalized for delivery in Omolbanin Hospital, Mashhad, Iran. Women who were admitted for delivery and satisfied and signed consent form entered in the study. First, demographic characteristic of subjects was recorded in questionnaire by two midwifes who were coworkers in this study. Then, before delivery, 4 mL of venous blood of women was taken for serum collection and PBMC separation. In addition, 2 mL of cord blood was taken and stored in a tube containing EDTA at the time of delivery. Data of gestational age, method of delivery, sex and birth weight of neonates, and presence or absence of fetal anomalies were recorded. Each day, collected samples were stored under cold chain storage condition during the transport to the Virology Laboratory of Ghaem Hospital, Mashhad, Iran. The prevalence of antibodies against HTLV1 was determined by ELISA using the commercial kit (Delaware Biotech Inc., USA). 

For positive subjects, DNA was extracted from PBMC using ficoll gradient method, and PCR was performed using HTLV1-specific primers (Table 1). Briefly, PCR mixture was prepared in two microtubes, and each one consisted of 10 pmol of each primer which LTR forward and reverse primers were added in one mixture, and Tax forward and reverse primers were used in another mixture. TBP forward and reverse primers were added in both reaction mixtures for amplifying a fragment of human TATAbox-binding protein as an internal control. Two micrograms of DNA sample, 1.5 mM MgCl_2_, 0.2 mM of each dNTP, and 1 *μ*L of Taq DNA polymerase (CinnaGen, Iran) were also added into each reaction mixture. Amplification was carried out for 41 cycles in AB Applied Biosystems Thermal Cycler. The PCR products were visualized on 2% agarose gels by Green Viewer staining. All six infants with positive PCR results were called again at the age of 9–12 months, and 2 mL of their peripheral blood was obtained. DNA was extracted from the PBMC using ficoll gradient method, and PCR was performed using HTLV1-specific primers as described above. The relation between HTLV1 antibody and demographic characteristic of the women and their neonates was analyzed by using Mann-Whitney test, Fisher's exact test, and software SPSS version 15. *P*  value < 0.05 was considered statistically significant.

## 3. Results 

In this study, 407 pregnant women participated, and most of them were 21–30 years old with a mean age of 26 years. HTLV1 antibodies were positive by ELISA in 6 (1.5%) subjects. Gestational age of all women who were positive for HTLV1 antibodies was term, and all of them had normal vaginal delivery (Table 2). So the age of all women with positive HTLV antibodies was 21–30 years (Table 2). The sex of their neonates who were born to HTLV1 antibodies-positive mothers was 4 boys and 2 girls, and their weight was in the normal range (Table 3). Statistical tests showed no significant relation between HTLV1 positivity and maternal age, gestational age, neonatal sex, and neonatal weight (*P* < 0.05). 

**Figure 1:**
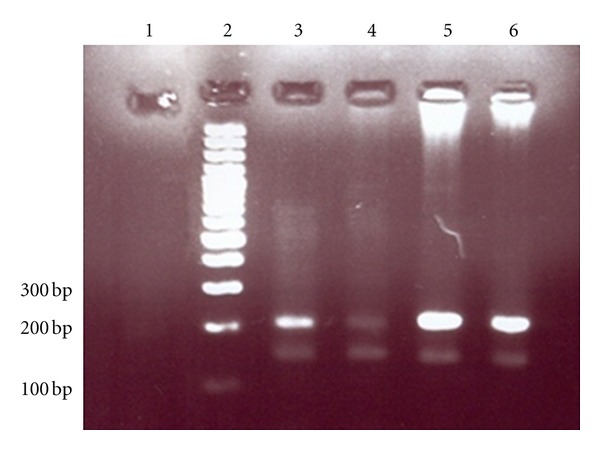
Gel electrophoresis of polymerase chain reaction product. Number 1 is a negative control of PCR, number 2 is 100 bp DNA size marker, and numbers 3, 4, 5, and 6 are HTLV1 positive samples which showed 222 bp and 149 bp fragments of HTLV1 LTR and human TATA-binding protein (internal control of PCR) genes, respectively.

**Figure 2:**
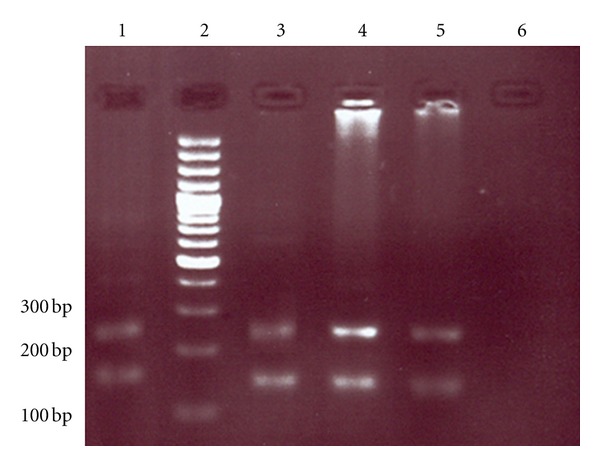
Gel electrophoresis of polymerase chain reaction product. Numbers 1, 3, 4, and 5 are HTLV1-positive samples which showed 256 bp and 149 bp fragments of HTLV1 TAX and human TATA-binding protein (internal control of PCR) genes, respectively. Number 2 is a 100 bp DNA size marker. Number 6 is a negative control of PCR.

In PCR method, all cord blood samples of neonates whose mothers were having seropositive HTLV1 antibodies were positive, and they showed 222 bp, 256 bp, and 149 bp fragments corresponding to HTLV1 LTR, HTLV1 TAX, and human TATA-binding protein (as an internal control), respectively (Figures 1 and 2). However, only one of them was PCR positive using HTLV1 TAX-specific primers (16.6%); this may be due to genetic changes in the infected viral genome.

**Figure 3:**
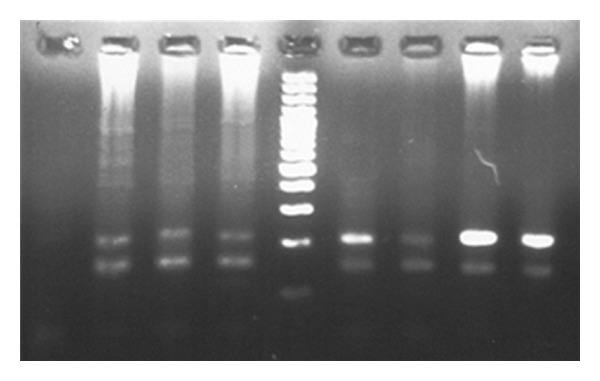
Gel electrophoresis of polymerase chain reaction product for the infants aged 9 to 12 months. Number 1 is a negative control of PCR, numbers 2, 3, 4, 6, 7, and 8 are HTLV1-positive samples which showed 222 bp and 149 bp fragments of HTLV1 LTR and human TATA-binding protein (internal control of PCR) genes, respectively. Number 5 is a 100 bp DNA size marker, and number 9 is a positive control of PCR.

**Figure 4:**
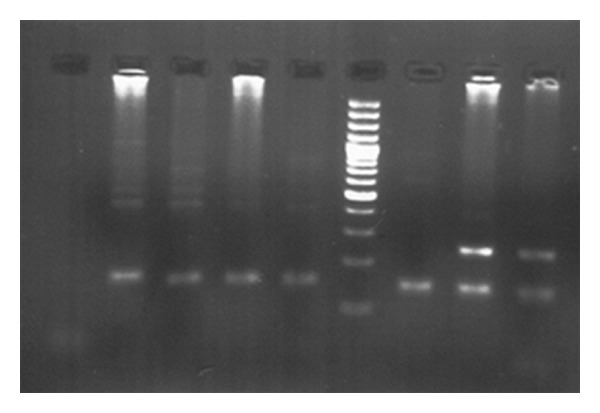
Gel electrophoresis of polymerase chain reaction product for the infants aged 9 to 12 months. Number 1 is a negative control of PCR, numbers 2, 3, 4, 5, and 7 are HTLV1 TAX negative samples, number 8 is HTLV1 TAX-positive sample which showed 256 bp fragment of HTLV1 TAX gene. Number 6 is a 100 bp DNA size marker and number 9 is a positive control of PCR. All samples (numbers 2, 3, 4, 5, 7, and 8) and positive control of PCR (number 9) showed a 149 bp fragment of human TATA-binding protein gene as an internal control of the PCR.

PCR results for infants aged 9 to 12 months are showed in Figures 3 and 4. All six infants showed positive results in PCR using LTR-specific primers (Figure 3); however, five of six infants had negative results in PCR using TAX-specific primers (Figure 4).

## 4. Discussion

Since Mashhad, in the northeast of Iran, has been suggested as an endemic area for human T-cell lymphotropic virus type 1 (HTLV1), we decided to determine the prevalence of HTLV1 in pregnant women and their neonates. In this study, 407 women who were admitted in the Omolbanin Hospital for delivery were examined for the presence of anti-HTLV1 which was positive in them; PCR test was performed for confirmation of proviral DNA in their peripheral mononuclear cells (PBMCs) and on samples of umbilical cord in their neonates. HTLV1 antibodies were positive by ELISA in 6 of the pregnant women (1.5%), and 16.6% of their neonates were positive. 

The mean age of participating pregnant women in our study was 26 years old and similar to pregnant women who were studied by Forbi in Nigeria and Themistocles in Brazil [11, 12]. There are areas in Japan, sub-Saharan Africa, the Caribbean, and South America where more than 1% of the general population is infected by HTLV. In some area in Taiwan, Iran, and Fujian (a Chinese province near Taiwan), the HTLV seroprevalence is 0.1–1% [13]. Hedayati-Moghaddam et al. showed that 35 of the 1511 serum samples were positive (2.3%) in Mashhad city [14]. In another study, Rafatpanah et al. that showed the prevalence of HTLV1 infection in Mashhad population was 2.12%, and they concluded that Mashhad is an endemic area for HTLV-I infection [15]. In another study, more than half of injected drug users living in the Central Prison of Mashhad were infected with HTLV1 [12]. Our data showed that the prevalence of HTLV1 in pregnant women was 1.5% which is similar to results of other scientists which were done in different years on the prevalence in general population in Mashhad.

Vertical transmission is one of HTLV1 transmission routes via placenta or at delivery and through breastfeeding. Katamine and colleagues study the possibility of intrauterine transmission as an alternative pathway to transmission via breast milk. They concluded that contamination of cord blood by maternal blood was the basis of viral load and lgA concentration, and cord blood proviral DNA is not a hallmark of intrauterine infection [16]. In a prospective study which was done by Nyambi et al. in Gabon, they evaluated the risk of HTLV1 transmission from mother to child, and none of the samples from umbilical cord blood and amniotic fluid were positive for the proviral DNA, but within 4 years, postnatal change in serum positivity was 17.5% [17]. In another study that was conducted by carles and colleagues, HTLV1 seroprevalence and associated risk factors were studied, and their children between 18 months to 12 years who were born to infected mothers with HTLV1 were studied. In this study, the relation between breastfeeding period and the level of HTLV1 antibodies and viral load was studied. The overall level of HTLV1 seroprevalence in maternal was 4.4%, and the relationship between its increasing risk and the number of pregnancies which is more than 6 and blood group RH negative factor was significant. So in 216 children who were born to HTLV1 infected mothers, 7.9% were seropositive for the virus although 180 of them had been breastfed. In this study the relationship between higher rate of HTLV1 antibody and proviral load in the mother and sex of neonate with seropositivity in children was significant, so in girls this was more common than in boys [18]. But results of our study showed that 100% of samples of umbilical cord blood of neonates who were born to HTLV1-positive mothers were positive. A retrospective study was done by Bittencourt and his colleagues in South Africa in 2002 and neonates who were born of HTLV1 positive mothers were investigated. They found, the transmission rate in breastfed neonates was 41% by PCR, and none of formula feeding neonates was positive [19]. In another study conducted by Toshitaka and colleagues in Japan, 16283 pregnant women were studied, and seropositive prevalence of HTLV1 in them was 5.4%, and serum change in their infants who were breastfed for less than 7 months was 3.8% and in formula-fed infants was 5.6% [20]. Sexual transmission is a significant source for acquisition of HTLV1 in adult, and overall 15–25% of children born to infected women become infected [21]. In our study, blood samples were prepared again from the six HTLV1-positive children at the age of 9–12 months, and PCR was done using the HTLV1-specific primers. Our data showed that HTLV1 LTR gene was amplified by PCR in every six children; while HTLV1 TAX gene was not amplified by the PCR in five of the six samples; this may be due to genetic changes in the viral genome as has been noted in the previous study [22].

## 5. Conclusion

Based on our data, the prevalence of HTLV1 antibodies in pregnant women was 1.5%, and vertical transmission rate to their neonates was 16.6%. As Mashhad in the northeastern area of Iran is endemic for HTLV1, further studies with larger samples and continuous surveillance are essential to monitor the virus infection in community.

## Figures and Tables

**Table 1:** Primers sequences for the PCR.

Target gene	Primers sequences	Fragment size
LTR	5′-CATAAGCTCAGACCTCCGGG-3′ 5′-GGATGGCGGCCTCAGGTAGG-3′	222 bp

Tax	5′-AGGGTTTGGACAGAGTCTT-3′ 5′-AAGGACCTTGAGGGTCTTA-3′	256 bp

TBP	5′-GTGAGAAGATGGATGTTGAGTTGC-3′ 5′-CAGATAGCAGCACGGTATGAGC-3′	149 bp

LTR: long terminal repeat; Tax: the HTLV transcriptional transactivator; TBP: human TATA binging protein; bp: base pair.

**Table 2:** The prevalence of HTLV1 antibodies in studied subjects according to the age distribution of women and their gestational age.

HTLV1 antibody	Negative	Positive	Total	*P* value
	No (%)	No (%)	No (%)	
Maternal age				
<21 years old	68 (17.0%)	0 (0.00%)	68 (16.7%)	
21–30 years old	248 (61.8%)	6 (100.0%)	254 (62.4%)	0.783
>30 years old	85 (21.2%)	0 (.0%)	85 (20.9%)	

Total	401 (100.0%)	6 (100.0%)	407 (100.0%)	

Gestational age				
Before term	23 (5.8%)	0 (.0%)	23 (5.8%)	
Term	347 (86.5%)	6 (100.0%)	353 (86.7%)	0.271
After term	31 (7.7%)	0 (0%)	31 (7.6%)	

Total	401 (100.0%)	6 (100.0%)	407 (100.0%)	

**Table 3:** The prevalence of HTLV1 in pregnant mothers and their newborns according to the sex and weight of newborns.

HTLV1 antibody	Negative	Positive	Total	*P* value
	No (%)	No (%)	No (%)	
Neonatal weight				
Lower than normal	16 (4.0%)	0 (0%)	16 (3.9%)	
Normal (2700–4000 gr)	370 (92.3%)	6 (100.0%)	376 (92.4%)	0.972
More than normal	15 (3.7%)	0 (.0%)	15 (3.7%)	

Total	401 (100.0%)	6 (100.0%)	407 (100.0%)	

Neonatal sex				
Male	202 (50.4%)	4 (66.7%)	206 (50.6%)	0.685
Female	199 (49.6%)	2 (33.3%)	201 (49.4%)	

Total	401 (100.0%)	6 (100.0%)	407 (100.0%)	

## References

[1] Folks TM, Khabbaz RF (1998). Retroviruses and associated disease in humans. *Topley & WilSon's Microbiology and Microbial Infections*.

[2] De Thé G, Bomford R (1993). An HTLV-I vaccine: why, how, for whom?. *AIDS Research and Human Retroviruses*.

[3] Hal Jenson B, Kliegman RM, Stanton BMD, Geme JSt, Schor N, Behrman RE (2001). Human T-lymphocyte virus(1 and 2). *Nelson Textbook of Pediatrics*.

[4] Blattner WA, Saxinger C, Riedel D (1990). A study of HTLV-I and its associated risk factors in Trinidad and Tobago. *Journal of Acquired Immune Deficiency Syndromes*.

[5] Mueller N, Okayama A, Stuver S, Tachibana N (1996). Findings from the Miyazaki Cohort study. *Journal of Acquired Immune Deficiency Syndromes and Human Retrovirology*.

[6] Kinoshita K, Hino S, Amagasaki T (1984). Demonstration of adult T-cell leukemia virus antigen in milk from three sero-positive mothers. *Gann*.

[7] Ikeda M, Fujino R, Matsui T (1984). A new agglutination test for serum antibodies to adult T-cell leukemia virus. *Gann*.

[8] Wiktor SZ, Alexander SS, Shaw GM (1990). Distinguishing between HTLV-I and HTLV-II by western blot. *Lancet*.

[9] Mirsadraee M, Kalantari MR, Saffari A, Mahmoudi M (2007). Association of HTLV1 infection and esophageal squamous cell carcinoma. *Journal of Gastrointestinal Cancer*.

[10] Miley WJ, Suryanarayana K, Manns A (2000). Real-time polymerase chain reaction assay for HTLV type I DNA viral load cell-associated. *AIDS Research and Human Retroviruses*.

[11] Forbi JC, Odetunde AB (2007). Human T-cell lymphotropic virus in a population of pregnant women and commercial sex workers in South Western Nigeria. *African Health Sciences*.

[12] Magalhães T, Mota-Miranda AC, Alcantara LC, Olavarria V, Galvão-Castro B, Rios-Grassi MF (2008). Phylogenetic and molecular analysis of HTLV-1 isolates from a medium sized town in northern of Brazil: tracing a common origin of the virus from the most endemic city in the country. *Journal of Medical Virology*.

[13] Verdonck K, González E, Van Dooren S, Vandamme AM, Vanham G, Gotuzzo E (2007). Human T-lymphotropic virus 1: recent knowledge about an ancient infection. *Lancet Infectious Diseases*.

[14] Hedayati-Moghaddam MR, Fathimoghadam F, Eftekharzadeh Mashhadi I, Soghandi L, Bidkhori HR (2011). Epidemiology of HTLV-1 in Neyshabour, Northeast of Iran. *Iranian Red Crescent Medical Journal*.

[15] Rafatpanah H, Hedayati-Moghaddam MR, Fathimoghadam F (2011). High prevalence of HTLV-I infection in Mashhad, Northeast Iran: a population-based seroepidemiology survey. *Journal of Clinical Virology*.

[16] Katamine S, Moriuchi R, Yamamoto T (1994). HTLV-1 proviral DNA in umbilical cord blood of babies born to carrier mothers. *Lancet*.

[17] Nyambi PN, Ville Y, Louwagie J (1996). Mother-to-child transmission of human T-cell lymphotropic virus types I and II (HTLV-I/II) in Gabon: a prospective follow-up of 4 years. *Journal of Acquired Immune Deficiency Syndromes and Human Retrovirology*.

[18] Carles G, Tortevoye P, Tuppin P (2004). HTLV1 infection and pregnancy. *Journal de Gynecologie Obstetrique et Biologie de la Reproduction*.

[19] Bittencourt AL, Sabino EC, Costa MC, Pedroso C, Moreira L (2002). No evidence of vertical transmission of HTLV-I in bottle-fed children. *Revista do Instituto de Medicina Tropical de Sao Paulo*.

[20] Oki T, Yoshinaga M, Otsuka H, Miyata K, Sonoda S, Nagata Y (1992). A sero-epidemiological study on mother-to-child transmission of HTLV-I in southern Kyushu, Japan.. *Asia-Oceania Journal of Obstetrics and Gynaecology*.

[21] Feigin RD, Cherry JD, Kaplan S, Demmler G Human retrovirus. *Textbook of Pediatric Infectious Disease*.

[22] Kazi A, Miyata H, Kamahora T, Kurokawa K, Katamine S, Hino S (1998). Deleted HTLV-1 provirus in cord-blood samples of babies born to HTLV-1-carrier mothers. *International Journal of Cancer*.

